# Development and Characterisation of an *in vitro* Model of Wallerian Degeneration

**DOI:** 10.3389/fbioe.2020.00784

**Published:** 2020-07-10

**Authors:** Heba Elsayed, Alessandro Faroni, Mohammad R. Ashraf, Judith Osuji, Lydia Wunderley, Ling Zhang, Hesham Elsobky, Mohamed Mansour, Ashraf S. Zidan, Adam J. Reid

**Affiliations:** ^1^Blond McIndoe Laboratories, Division of Cell Matrix Biology and Regenerative Medicine, School of Biological Sciences, Faculty of Biology, Medicine and Health, The University of Manchester, Manchester Academic Health Science Centre, Manchester, United Kingdom; ^2^Department of Neurosurgery, Mansoura University Hospitals, Mansoura, Egypt; ^3^Division of Cellular and Molecular Function, School of Biological Sciences, Faculty of Biology, Medicine and Health, The University of Manchester, Manchester Academic Health Science Centre, Manchester, United Kingdom; ^4^College of Polymer Science and Engineering, Sichuan University, Chengdu, China; ^5^Mansoura University Hospital, Mansoura, Egypt; ^6^Department of Plastic Surgery & Burns, Wythenshawe Hospital, Manchester University NHS Foundation Trust, Manchester Academic Health Science Centre, Manchester, United Kingdom

**Keywords:** Wallerian degeneration, Schwann cells, nerve injury, myelin degradation, neurotrophic factors, *in vitro* model, adipose-derived mesenchymal stem cells

## Abstract

Following peripheral nerve injury, a sequence of events termed Wallerian degeneration (WD) takes place at the distal stump in order to allow the regenerating axons to grow back toward the target organs. Schwann cells (SCs) play a lead role in this by initiating the inflammatory response attracting macrophages and immune cells, as well as producing neurotrophic signals that are essential for nerve regeneration. The majority of existing research has focused on tools to improve regeneration, overlooking the critical degeneration phase. This is also due to the lack of *in vitro* models recapitulating the features of *in vivo* WD. In particular, to understand the initial SC response following injury, and to investigate potential interventions, a model that isolates the nerve from other systemic influences is required. Stem cell intervention has been extensively studied as a potential therapeutic intervention to augment regeneration; however, data regarding their role in WD is lacking. Thus, in this study we describe an *in vitro* model using rat sciatic nerve explants degenerating up to 14 days. Characterisation of this model was performed by gene and protein expression for key markers of WD, in addition to immunohistochemical analysis and electron microscopy. We found changes in keeping with WD *in vivo*: upregulation of repair program protein CJUN, downregulation of myelin protein genes and subsequent disorganisation and breakdown of myelin structure. As a means of testing the effects of stem cell intervention on WD we established indirect co-cultures of human adipose-derived mesenchymal stem cells (AD-MSC) with the degenerating nerve explants. The stem cell intervention potentiated neurotrophic factors and C*jun* expression. We conclude that our *in vitro* model shares the main features of *in vivo* WD, and we provide proof of principle on its effectiveness to study experimental approaches for nerve regeneration focused on the events happening during WD.

## Introduction

Treatment options following peripheral nerve injuries are dependent on surgical interventions which have demonstrated that the perfect microsurgical repair of injured nerve stumps cannot alone restore prior function to the patient (Palispis and Gupta, 2017). A multitude of research has therefore focused on enhancing regeneration of peripheral nerves, perhaps neglecting the pre-requisite of distal stump degeneration to lay the groundwork for subsequent regeneration. WD is orchestrated by SC plasticity (Boerboom et al., 2017) with the ability to dedifferentiate from a myelinating phenotype to a repair phenotype. This process involves upregulation of genes such as *Cjun* and *P75* encoding proteins responsible for proliferation, migration, adhesion and systemic inflammatory response activation (Jessen and Arthur-Farraj, 2019). SCs initiate the inflammatory response to increase the number of macrophages and monocytes in the distal stump; moreover, both act to clear myelin debris in order to pave the way for the regenerating growth cone to navigate toward the distal stump (Dubovy, 2011). Therefore, it is logical that the degenerative phase of clearing axon-myelin debris could be targeted for therapeutic intervention in order to accelerate or improve efficiency of the process and ultimately promote regeneration.

Models of WD used in research are limited by poor characterisation, failure to isolate the critical SC response from the systemic response and lack of potential to translate into human tissue models. Recent insightful studies have used injured *ex vivo* nerve models of WD in genetic knockout mice to identify mechanisms underpinning autophagic action of SCs (Gomez-Sanchez et al., 2015; Jang et al., 2016). Furthermore, macrophages and SCs are known to act synergistically in the degradation and removal of myelin (myelinophagy) following injury (Gaudet et al., 2011; Rotshenker, 2011). In order to understand the capacity of the resident cells to regulate myelinophagy independent of the systemic response, it is necessary to characterise a model which isolates the injured nerve immediately following injury. This model would also permit investigation of interventions in the degenerative phase following nerve injury.

Perhaps the most promising experimental intervention for regeneration is the use of stem cells; however, nothing is known of their impact on WD (Dezawa et al., 2001; Kingham et al., 2007). AD-MSCs have been demonstrated to improve neuronal regeneration *in vitro* and *in vivo* models following chemical stimulation into a Schwann-like phenotype (dASC) (Faroni et al., 2013); however, survival *in vivo* after nerve injury has been demonstrated to be less than a few weeks, therefore their therapeutic window spans only the initial regenerative response (Faroni et al., 2016). As such, it stands to reason that AD-MSC and dASC may impact WD as well as the regenerative response and perhaps through a similar mechanism of growth factor (BDNF and NGF) production. In this work, we establish and characterise an *in vitro* model of WD to determine ultrastructural and histological morphology, alongside a timeline of important gene and protein expression changes over 14 days. We subsequently use this model to test the effect of AD-MSCs and dASC intervention on SC functions during WD.

## Materials and Methods

### Establishing an *in vitro* Explant Model

All the animal work was performed in accordance with the UK Animal Scientific Procedures Act 1986. Following CO_2_ euthanasia and cervical dislocation (S1) the hind limbs of adult 200–300 g Sprague Dawley rats were carefully dissected in surgical planes to expose the whole course of sciatic nerve up to its origin. Blunt dissection freed the nerve from underlying fascia and muscle before being divided and placed into Hanks balanced salt solution (HBSS, Sigma-Aldrich). Four rats (eight sciatic nerves) were used per experiment. Nerves were de-sheathed (removal of the epineurium) unless otherwise specified under a microscope at 20× magnification, and subsequently divided into smaller pieces (nerve explants) of 5–10 mm size, using sharp micro-scissors. For all experiments, day 0 time points were processed first and frozen at −80°C or fixed immediately, whereas for days 1, 3, 5, 7, and 14, the nerve explants were placed in six well plates containing 2 ml of low glucose Dulbecco’s Modified Eagle’s Medium (DMEM; Sigma-Aldrich, United Kingdom) supplemented with 10% (v/v) of FBS (LabTech, Uckfield, United Kingdom), 1% (v/v) of penicillin–streptomycin solution (PS; Sigma-Aldrich, United Kingdom), and 2 mM L-glutamine (GE Healthcare UK, Little Chalfont, United Kingdom). No media change was performed in the first 7 days of culture; however, for explants to be collected at day 14, a single media change was performed at day 7. All explants were incubated in 5% CO_2_ at 37°C, frozen or fixed at their respective time points, and stored at −80°C until further processing.

### Transmission Electron Microscope (TEM)

For electron microscopy studies, nerve explants (not de-sheathed) at each time point were pinned to a plastic strip with sterile needles and fixed by immersing in a 2% (w/v) PFA and 2% (v/v) glutaraldehyde solution for 24 h, transferred into PBS, and refrigerated until all time points were collected. The samples were then processed by University of Manchester Electron Microscopy Facility as ultrathin nerve cross sections for TEM imaging. The nerve samples were fixed for an hour in 1% osmium tetroxide and 1.5% potassium ferrocyanide in 0.1 M cacodylate buffer, followed by another 24 h in 1% uranyl acetate in water. The sample was then dehydrated in an ethanol series with 812 resin (TAAB laboratories), before polymerization for 24 h at 60°C. Samples were cut with the Reichert Ultracut Ultra-Microtome as ultra-thin sections. These were visualised with FEI Tecnai 12 Biotwin microscope at 100 kV accelerating voltage, and snapshots saved with Gatan Orius SC1000 CCD camera.

### Immunohistochemistry (IHC)

In order to preserve the fine structure of the nerve, samples were not de-sheathed when processed for IHC, with the exception of the DAPI count experiment (Figure 2B). Nerve explants were pinned to a plastic strip to straighten up with minimal tension and were fixed for 24 h by immersing in 4% (w/v) PFA, followed by three serial changes with 15% (w/v) sucrose and 0.1% (w/v) sodium azide in PBS. Samples were refrigerated until embedding in optimal cutting temperature (OCT) cryo-compound (Klinipath), after which they were stored at −80°C. The blocks were then cut longitudinally at University of Manchester histology facility by using cryostat (Leica ASP300). Sections were cut at 5 um and caught in between the microtome blade and glass. The sections were transferred to pre-heated Superfrost Plus slides (VWR International) and then incubated overnight at 37°C. Slides were then stored at −80°C until staining procedure. On the staining day, the slides were submerged in 0.2% Triton X-100/PBS for 1 h at RT then a blocking serum containing normal goat or donkey serum solution (both 5:100; Sigma-Aldrich, Poole, United Kingdom) was added for 1 h at RT after 2 × PBS washes 5 min each. The slides were incubated with the primary antibody [P0 Abcam (ab183868) 1:200], [MBP Millipore (MAB384-1ML) 1:200] βIII Tubulin [βIIIT Sigma (ab6046) 1:200] and CD68 [AbD serotec (MCA1957GA) 1:200] overnight at 4°C followed by two PBS washes and secondary antibody incubation the following day for 1 h at RT [goat/monkey anti-rabbit and goat/monkey anti-mouse (488 for green and 568 for red) (Life Technologies, Thermo Fisher Scientific)] in dark.

In desheathed nerve segments, explants were treated as above with the addition of three PBS washes for 5 min before mounting with Vectashield (Vector Laboratories, United Kingdom) containing 4′,6-diamidino-2-phenylindole (DAPI) for nuclear staining. This permitted the quantification of cells which are presumed to be predominantly SCs.

For FluoroMyelin staining: the slides were washed with PBS for 20 min and stained with green FluoroMyelin dye [1:300 in PBS, Molecular Probes, United Kingdom (F34651)] for 20 min at RT. Sections were washed 3 × 5 min with PBS before mounting with aqueous media containing DAPI (Vector Laboratories, United Kingdom).

Images were acquired using a fluorescence microscope (Olympus BX60, Southend-on-Sea, United Kingdom), and processed with ImageJ imaging software (Version 1.52a, National Institutes of Health NIH, Bethesda, MD, United States).

### RNA Extraction

The RNeasy Lipid Tissue Mini Kit (QIAGEN, United Kingdom) was used to extract purified RNA from the nerve samples according to the manufacturer’s protocol. Briefly, each of the frozen nerve samples were placed in 5 ml Falcon Polypropylene Rounded-Bottom Tube containing 1 ml QIAzol Lysis Reagent (QIAGEN, United Kingdom). A TissueRupter tissue homogeniser (QIAGEN, United Kingdom) was used to homogenise the nerve explants on ice. After 5 min, the sample was transferred to a 1.5 ml Eppendorf tube to which 200 ul of chloroform was added (Sigma-Aldrich). The tube was then vortexed for a few seconds, allowed to sit for 2–3 min, before being centrifuged at 12,000 × *g* for 15 min at (4°C). The aqueous phase was isolated and one volume of 70% ethanol (Fisher Chemical) was added; following gentle pipetting, the resulting solution was transferred to RNeasy Mini spin column (QIAGEN) in 2 ml collection tube and centrifuged for 30 s at 13,000 rpm at RT. Columns were then washed with Buffer RW1, and twice with Buffer RPE. Finally, the column was placed into a 1.5 ml Eppendorf tube, and 30 ul of RNase free water (QIAGEN) was added directly to the column to elute RNA with a 1 min centrifugation at 13,000 rpm at RT.

The RNA concentration was determined by spectrophotometric analysis with a NanoDrop ND-100 (Thermo Fisher Scientific, Waltham, MA, United States).

### Real Time qPCR (RT-qPCR)

1 μg of each sample was reverse transcribed using the RT2 First Strand Kit (Qiagen), according to the manufacturer’s instructions. Briefly, 2 μl buffer GE (QIAGEN) was added to 1 μg of RNA diluted in 8 μl RNase free water and the resulting mixture was incubated for 5 min at 42°C to eliminate genomic DNA, before being cooled down to 4°C for 20 min. 10 μl of Reverse Transcription mix was added to the sample according to manufacturer’s guide. Each sample was then incubated at 42°C for 15 min using the PTC-200 Peltier Thermal Cycler (MJ Research). The reaction was stopped by incubating the samples at (95°C) for 5 min, before being cooled down to 4°C for 20 min. 91 ul of RNase free water was finally added to the resulting reverse transcribed cDNA sample.

RT-qPCR was performed using a Corbett Rotor Gene 6000 (Qiagen) and RT2 SYBR Green qPCR Mastermix (Qiagen) as follows: hot start for 10 min at 95°C, followed by 40 cycles of 15 s at 95°C, annealing for 30 s at 55°C, and extension for 30 s at 72°C. Melting curve was obtained as follow: 95°C for 1 min, 65°C for 2 min, and a gradual temperature increase from 65°C to 95°C at 2°C/min. All of the primers for the genes of interest and for the housekeeping gene were obtained from Sigma-Aldrich (listed in Table 1). Data were normalised for the housekeeping gene (GAPDH or 18S) and the ΔΔCt method was used to determine the fold changes in gene expression. Data is presented as mean ± standard error of the mean (SEM, error bars) and analysed by one-way ANOVA (Tukey’s multiple comparison test).

**TABLE 1:** Primer sequences used in PCR studies.

Gene	Forward	Reverse
*Sox10*	TGCCTAGGGACCTGCTTCAA	GGGTGAAAGGATCAGAGTGTCC
*Cjun*	GACCTTCTACGACGATGC	CAGCGCCAGCTACTGAGGC
*Oct6*	TCCGACGACCTGGAGCAG	CGAAGCGGCAGATGGTGG
*Krox20*	ACTGCTACCCCCTACAATCCGC	GAACCTCCTGTCGCAACCCTCT
*S100*	TCCATCAGTATTCAGGGAGAGA	TCCATCACTTTGTCCACCACT
*P75*	CACGACCAGCAGACCCATA	GCCAGATGTCGCCAGGTAT
*Nestin*	CCGGGTCAAGACGCTAGAAGA	CTCCAGCTCTTCCGCAAGGTTGT
*Ngf*	AAGGACGCAGCTTTCTATCC	CTATCTGTGTACGGTTCTGCC
*Bdnf*	AAGTCTGCATTACATTCCTCGA	GTTTTCTGAAAGAGGGACAGTTTAT
*Gdnf*	CAGTGACTCCAATATGCCCGA	TCGTAGCCCAAACCCAAGTC
*P0*	CCTGCTCTTCTCTTCTTTG	CACAGCACCATAGACTTC
*Mbp*	CGCATCTTGTTAATCCGTTCTAAT	GAGGGTTTGTTTCTGGAAGTTTC
*Pmp22*	TCCTGTTCCTTCACATCG	TGCCAGAGATCAGTCCTG
*Trka*	CCCCATCCCTGACACTAACA	GAGCAGCGTAGAAAGGAAGAG
*Gapdh*	CCGTATCGGACGCCTGGTTA	CCGTGGGTAGAGTCATACTGGAAC
*18S*	GGATCCATTGGAGGGCAAGT	ACGAGCTTTTTAACTGCAGCAA

### Western Blot

Nerve explants were homogenised using a TissueRuptor (QIAGEN) in radio-immunoprecipitation assay (RIPA Sigma, United Kingdom) buffer (800 μl each) supplemented with a cocktail of protease and phosphatase inhibitors (Thermo Scientific, Loughborough, United Kingdom), and left for 30 min on ice. Following centrifugation at 14,000 rpm for 20 min at (4°C), proteins were quantified using Pierce^TM^ BCA Protein Assay Kit (Thermo Scientific, Waltham, MA, United States) according to the manufacturer’s instruction. Protein samples were boiled in RIPA buffer and Laemmli sample buffer (×6) for 5 min at 100°C. An equal amount of the protein of interest (listed in Table 2) was loaded and run in sodium dodecyl sulphate polyacrylamide gels (SDS-PAGE) using Tris-glycine running buffer [25 mM Tris, 190 mM glycine, 0,08% (w/v) SDS]. The proteins were then transferred onto a nitrocellulose membrane (GE Healthcare Life Science, Amersham, Germany) in transfer buffer [25 mM Tris-base; 192 nM glycine, 20% (v/v) methanol]. The membranes were blocked for 1 h in a Tris-buffer saline (TBS) - Tween Solution containing 5% non-fat dry milk. Membranes were incubated overnight with primary antibodies (details in Table 2) diluted in blocking buffer or 5% bovine serum albumin (BSA) (Sigma-Aldrich, United Kingdom). Washes were done using TBS-Tween followed by 1 h incubation of the secondary antibody horseradish peroxidase (HRP) conjugated against rabbit or mouse (Cell Signaling, Hitchin, United Kingdom) for chemiluminescence detection. For housekeeping protein (GAPDH) membranes were stripped with a glycine solution (100 mM, pH 2.9; Sigma-Aldrich, United Kingdom) for 15 min at RT and re-blocked again as before, prior to further blotting. Membranes were exposed to SuperSignal West Pico Chemiluminescent Substrate (Thermo Scientific, Waltham, MA, United States), and the signal was analysed by densitometry using ImageJ 64 imaging software (Version 1.52a, National Institutes of Health NIH, United States). Western blot densitometry data of all proteins were normalised for the levels of GAPDH for each time point and used as a loading control.

**TABLE 2:** Antibodies and experimental conditions used for Western Blot analyses.

Protein name	Company	Gel%	Amount	Transfer	1ry Ab	2ry Ab
BDNF Rabbit polyclonal	Alomone Labs (ANT-010)	15%	20 μg	1.5 h at RT 30 MV	1:200 in 3% BSA	1:2000 in 3% BSA
NGF Rabbit polyclonal	Alomone Labs (AN-240)	15%	20 μg	1.5 h at RT 30 MV	1:400 in 3% BSA	1:2000 in 3% BSA
CJUN Rabbit polyclonal	Abcam, United Kingdom (ab31419)	15%	20 μg	1.5 h at RT 30 MV	1:500 in 5% milk	1:2000 in 5% milk
MBP Mouse monoclonal	Millipore, United States (MAB384-1ML)	15%	10 μg	Overnight at 4°C 30 MA	1:500 in 5% milk	1:2000 in 5% milk
P0 Rabbit monoclonal	Abcam, United Kingdom (ab183868)	15%	5 μg	Overnight at 4°C 30 MA	1:5000 in 5% milk	1:10000 in 5% milk
P75 Rabbit monoclonal	Abcam, United Kingdom (ab52987)	10%	20 μg	1 h at 80 V	1:500 in 5% milk	1:2000 in 5% milk
KROX20 Rabbit Polyclonal	Proteintech, United States (13491-1-AP)	10%	20 μg	1 h at 80 V	1:500 in 5% milk	1:2000 in 5% milk
GAPDH Rabbit polyclonal	Proteintech, United States (10494-1-AP)				1:5000 in 5% milk	1:4000 in 5% milk

### Harvest and Culture of Human AD-MSCs

Adipose tissue samples were collected from patients undergoing reconstructive breast surgery at Wythenshawe Hospital, Manchester University NHS Foundation Trust, Manchester, United Kingdom. Patients were fully consented and procedures approved by the National Research Ethics Committee (NRES 13/SC/0499). All the samples were derived from subcutaneous abdominal fat. All patients were females aged 45–65 years and otherwise fit and healthy. AD-MSCs were obtained according to previously described protocol (Kingham et al., 2007). The adipose tissue was cut into small pieces then minced with razor blade and digested enzymatically using 0.2% (w/v) collagenase (Life Technologies, Paisley, United Kingdom) at 37°C for 45 min under constant agitation. The resulting tissue was filtered through a vacuum-assisted 100 μm nylon mesh (Merck Millipore UK, Watford, United Kingdom), followed by the addition of an equal amount of alpha-modified Minimum Essential Eagle’s medium (aMEM) (Sigma-Aldrich, Poole, United Kingdom) supplemented with, 10% (v/v) FBS (LabTech, Uckfield, United Kingdom), 2 mM L-glutamine (GE Healthcare UK, Little Chalfont, United Kingdom), and 1% (v/v) penicillin–streptomycin solution. After centrifugation at 400 × *g* for 10 min, the SVF was pelleted. 1 ml of Red Blood Cell Lysis Buffer (Sigma-Aldrich) was added for 1 min followed by the addition of 15 ml of fresh aMEM and further centrifugation at 400 × *g* for 10 min. The resulting SVF pellet was plated in T75 at 37°C and 5% CO_2_ with media change twice to three times a week and split when confluent. Characterisation was done according to our previously published protocol (Faroni et al., 2016). Passage 2 cells were used for co-culture experiments involving AD-MSC.

### Stimulation of AD-MSC to a Schwann-Like Phenotype dASC

As previously described (Kingham et al., 2007), passage 2 AD-MSC were plated at 20 to 30% confluency and treated with 1 mM b-mercaptoethanol (Sigma-Aldrich) for 24 h, prior to 72 h of exposure to 35 ng/mL all-*trans*-retinoic acid (Sigma-Aldrich). The differentiation media (d-aMEM) was then added ([aMEM) + 14 μM forskolin (Sigma-Aldrich) + 5 ng/mL platelet-derived growth factor (Peprotech EC, London, United Kingdom) + 192 ng/mL glial growth factor-2 (GGF-2) (Acorda Therapeutics, Ardsley, NY, United States) + 10 ng/mL basic fibroblast growth factor (Peprotech EC)]. Cells were split at the fourth and tenth days of differentiation, and cells were considered differentiated (dASCs) after 14 days, as previously reported (Kingham et al., 2007). Cells at passage 4 to 6 were used in co-culture experiment involving dASCs.

### Co-culture Experiments

Twenty-four hours before nerve explants were harvested, both AD-MSC and dASCs cells from confluent flasks were collected and counted using a Scepter 2.0 (Merck-Millipore). 100,000 cell per well were plated in 24-well plates (three wells for each time point) and 500 μl of either aMEM or d-aMEM media was used for each well.

Nerve explants were collected from four Sprague Dawley rats as described above, with 15–20 nerve explants collected for each condition. All the nerves were de-sheathed under the microscope and cut into 5–10 mm length. Experimental groups were as follow: D0 healthy nerve explants (D0 c), D3 degenerated nerve explants cultured without cells (D3 c), D3 nerves co-cultured with AD-MSC (uASCs) and D3 nerve explants cultured with dASCs (dASCs).

Using an insert with a 0.4 um porous membrane (Greiner Bio-One), the nerve explants were inserted into the 24 well plate containing the plated cells after a media change and HBSS wash. 1200 μl of DMEM media was added to both the cells and the nerves (across the permeable membrane). The well insert ensured that no direct contact between AD-MSC and explants occurred. The co-cultures were kept in the incubator at 37°C at 5% CO_2_ for 3 days without a media change. The nerve explants were collected at each time point and processed for either gene or protein expression as described above.

### Statistical Analysis

Data analysis were performed using GraphPad Prism 5 (7.0, GraphPad Software Inc., La Jolla, CA, United States). PCR data were presented as mean ± standard error of the mean (SEM, error bars) by one-way ANOVA (Tukey’s multiple comparison test) (*p < 0.05, **p < 0.01, ***p < 0.001, ****p < 0.0001).

## Results

### TEM Highlights Morphological/Structural Features of Degenerating Nerves in the *in vitro* WD Model

**FIGURE 1:**
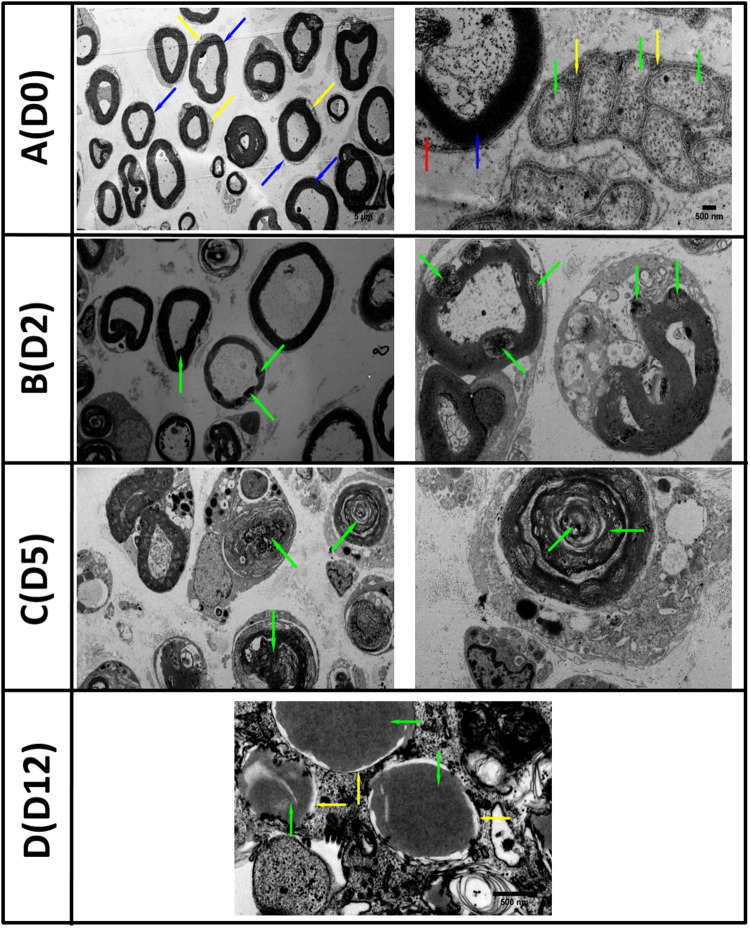
TEM images of *in vitro* Wallerian degeneration model showing ongoing degeneration of the nerve axons and myelin. **(A)** (D0 left) Healthy rat sciatic nerve at day zero (D0) showed preserved myelin structures (blue arrows) and surrounded by Schwann cells cytoplasm (yellow arrow). **(A)** (D0 right) Nerve at higher magnification with regular myelin lamellae (blue arrow) and Schwann cell nucleus (red arrow). Multiple unmyelinated axons (green arrow) surrounded by Remak Schwann cell (yellow arrow) grouped in Remak bundles were also observed**. (B)** (D2 left) The second day (D2) post transection, the Wallerian Degeneration (WD) was evidenced by the appearance of multiple vacuoles within the myelin structures (green arrows) with the collapse of myelin evident at higher magnification **(B)** (D2 right). **(C)** Five days (D5) post *in vitro* WD we observed the disappearance of axonal structures and abundance of myelin debris within Schwann cell cytoplasm (green arrows). **(D)** Twelve days (D12) post transection and WD, the axons were replaced with amorphous material (green arrows) and myelin structures disappeared (yellow arrows) (Scale bars as in the top row are 5 μm on the right and 500 nm on the left).

In order to assess WD *in vitro*, we performed TEM analysis of degenerating explants at day 0, 2, 5, and 12 after transection and degeneration *in vitro*. Transmission electron micrographs showed degeneration of both axons and myelin with loss of nerve structure and disorganisation (Figure 1). Day 0 (D0) healthy non-injured sciatic nerve (Figure 1A) (left panel) showed preserved myelin structures (blue arrows) and cytoplasm of surrounding SCs was visible (yellow arrow). At higher magnification (right panel) we observed regular myelin lamellae (blue arrow) with SC nucleus (red arrow), alongside multiple unmyelinated axons (green arrow) surrounded by Remak SC (yellow arrow) to form Remak bundles. Two days after the *in vitro* degeneration (D2) the injured sciatic nerves contained multiple vacuoles within the myelin structure with evidence of early disorganisation and myelin collapse, accompanied by gradual loss of lamellar myelin structure (highlighted by green arrows at low (left) and high (right) magnification, Figure 1B). At day 5 (D5) post injury we observed the disappearance of some axons and the presence of multiple myelin debris within SC cytoplasm (green arrows), at low (left) and high (right) magnification (Figure 1C). After 12 days (D12) of WD induction *in vitro*, the nerve structure was compromised, myelin was unfolded or completely degenerated (yellow arrows), and axonal structure was replaced by amorphous material (green arrows) denoting the complete degeneration of the nerve sections (Figure 1D).

**FIGURE 2:**
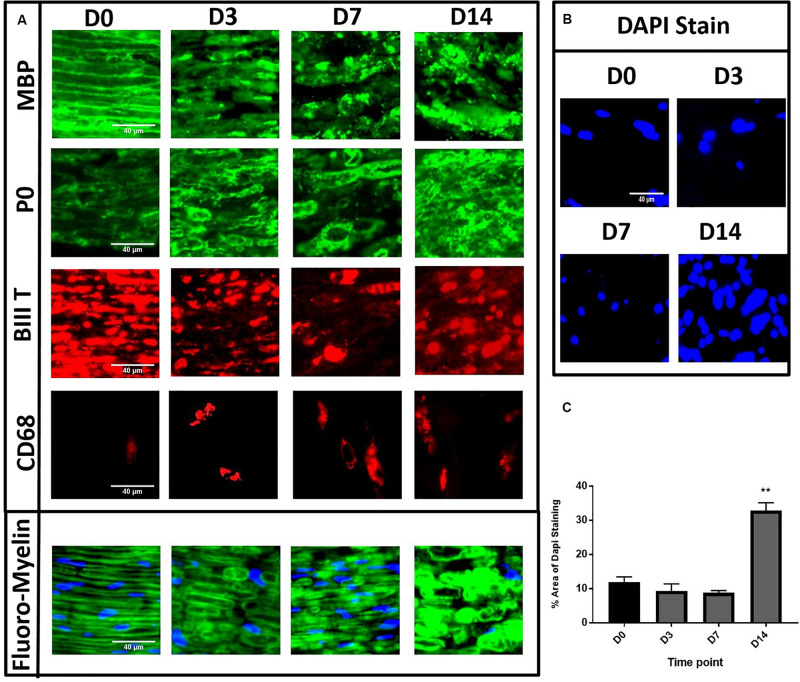
Immunohistochemistry of *in vitro* model of degeneration showing degenerative changes to both myelin lipids, myelin proteins and axons. Panel **(A)** showed MBP/βIIIT/P0 staining with evident degeneration at D14 and Fluoro-Myelin staining with lipid degeneration. (D0) Compact homogenous organised myelin and axons resembling normal nerve. (D3) Loosening and disorganisation of myelin with multiple discontinuity. (D7) Continued loosening and disorganisation with greater disruption of myelin structure. (D14) Consolidated mass with loss of architecture. CD68 staining with gradual increase in numbers of cells after degeneration induction. **(B,C)** Gradual increase of % area of DAPI staining in desheathed explants at different time points showing significant increase at D14. (D0 is control.) (**P < 0.001) Scale bar for MBP, P0, βIIIT, Fluoro-Myelin and DAPI is 40 μm. Scale bar for CD68 is 200 μm.

### The *in vitro* Explant Model Shows Typical Immunohistochemical Features of WD

To further investigate the extent of WD in our *in vitro* model, we performed immunohistochemical analyses for myelin proteins, axonal structures and macrophage markers. The nerve explants were collected and fixed at different time points following *in vitro* WD (D0, 3, 7, and 14). Embedded nerves were cut in longitudinal sections and stained with myelin protein antibodies MBP and P0, FluoroMyelin (a non-specific stain for myelin lipids), axonal marker βIII tubulin as well as macrophage marker CD68. We observed consistent patterns of distribution of myelin and axonal structures at the different time points in three separate experiments. Healthy nerve at D0 (Figure 2A) showed compact myelin structures with complete homogeneity of the MBP and P0 staining (green), with lamellar organisation of myelin proteins and associated axons (βIII tubulin, red staining). At D3 post WD induction we observed early loosening and subtle disorganisation of both myelin lamellae (green) and corresponding axons (red) with appearance of multiple myelin discontinuity. With the progression of degeneration at D7, we found further loosening of the myelin structures and disorganisation, which progressed to a complete loss of tissue architecture at D14 post-harvest (Figure 2A). Interestingly, we noticed a progressive increase in the number of CD68 positive cells from D0 to D14, a macrophage marker, in non-desheathed nerves (Figure 2A), compared to nearly complete absence in de-sheathed nerve sections (results not shown), suggesting that macrophages only migrate within the nerve after WD is induced. Finally, we quantified the number of nuclei present in the nerve sections, stained by DAPI as an indication of cell density within the explant. The area of DAPI staining (%) in desheathed nerves was calculated from three independent experiments and showed a significant three-fold increase in the number of cells at D14 in the *in vitro* model (*p* < 0.01) compared to D0 intact nerve (Figures 2B,C).

### Gene Expression Level of WD Markers Indicate Progression of Degeneration in the *in vitro* Model

In order to assess the efficacy of the *in vitro* model to recapitulate WD, gene expression for key transcription factors, SC markers, neurotrophic markers and myelin genes was performed on D0 to 7 samples of degenerating nerves.

**FIGURE 3:**
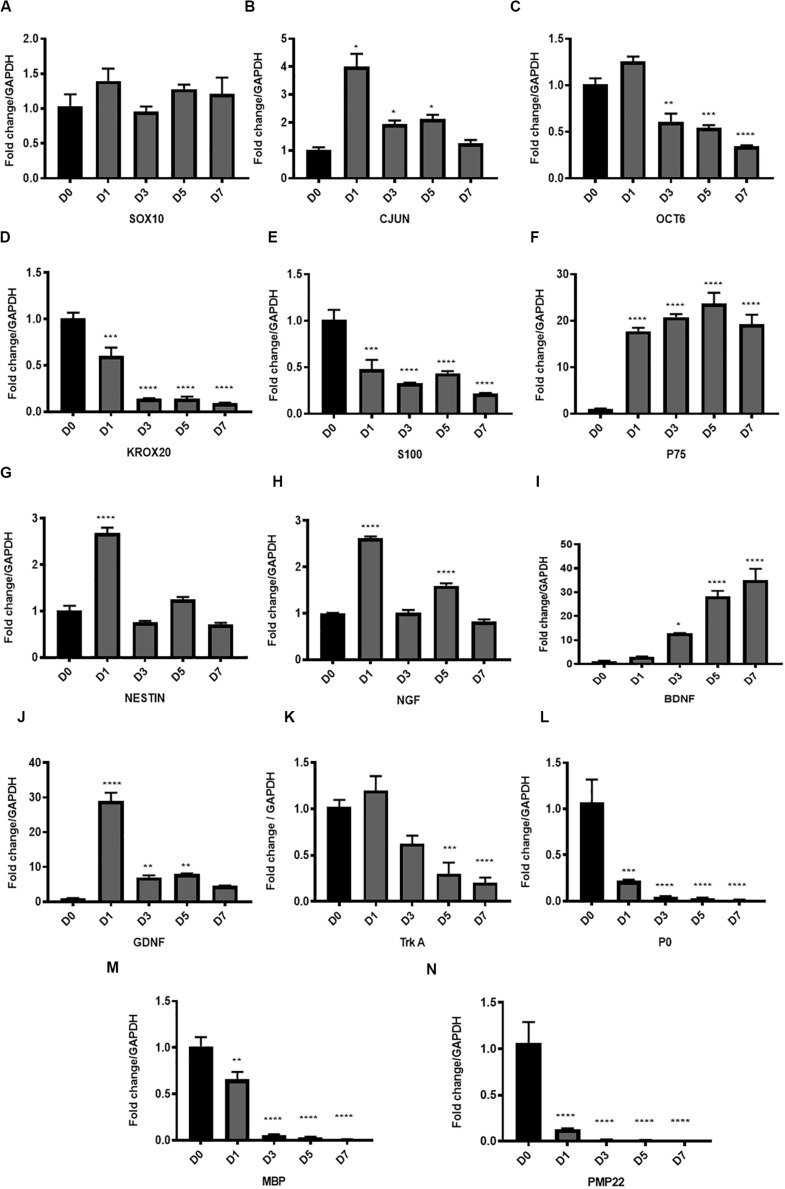
Gene expression of key transcription factors, Schwann cell markers, myelin proteins and neurotrophic factors in the *in vitro* model of Wallerian degeneration. **(A)** No significant change in *Sox10* gene expression levels after *in vitro* degeneration. **(B)** Significant upregulation of *Cjun* transcription factor after injury mostly at D1 compared to D0 healthy nerve with return to baseline levels at D7. **(C)** Transcription factor *Oct 6* expression after WD induction showed significant downregulation at D3, 5 and 7 post transection. **(D)** Transcription factor *Krox20* showed significant progressive downregulation in its expression levels with *in vitro* degeneration. **(E)** SC marker *S100* after *in vitro* WD showed significant downregulation relative to D0 (control healthy nerve). **(F)** *P75* after *in vitro* WD showed significant persistent upregulation. **(G)** Neuronal marker and intermediate filament *Nestin* showed transient significant upregulation at D1 post-harvest followed by return to baseline levels at later time points. **(H)** Expression of *Ngf* after *in vitro* WD was biphasic with significant surge of its expression at D1 and D5 compared to D0 healthy nerve. **(I)** Neurotrophic factor *Bdnf* showed significant continuous upregulation after *in vitro* WD in later time points. **(J)** Neurotrophic factor *Gdnf* showed significant early upregulation at D1 followed by gradual reduction at D3, 5 and D7 post-harvest. **(K)** The *Ngf* receptor *Trka* showed gradual downregulation of its gene expression in *in vitro* WD reaching significant reduction at D7 compared to D0 (control healthy nerve). **(L)** Gene expression of *P0* after WD induction showed early significant downregulation relative to D0 (control). **(M)** Gene expression of *Mbp* showed early significant downregulation relative to D0 (control). **(N)** Gene expression of *Pmp 22* after WD induction showed significant downregulation relative to D0 (control). Data are expressed as log2 fold change in D1, 3, 5 and 7 relative to D0 healthy nerve control sample. Data is presented as mean ± SEM (error bars) by one-way ANOVA (Tukey’s multiple comparison test) (*P < 0.05, **P < 0.01, ***P < 0.001, ****P < 0.0001).

We analysed key transcription factors for SC function. We did not observe any change in *Sox10* gene expression levels after *in vitro* degeneration (Figure 3A). Conversely, there was significant upregulation of *Cjun* post WD induction, especially at D1 compared to D0 healthy nerve (*P < 0.05), although levels returned at baseline levels by D7 (Figure 3B). *Oct6* and *Krox20* genes were significantly and progressively downregulated with *in vitro* degeneration, reaching minimal levels at D7 following injury (****P < 0.0001 vs. D0) (Figures 3C,D).

Amongst the SC markers, we found significant downregulation of *S100* expression levels (***P < 0.001 and ****P < 0.0001 at D1 and D3, 5, 7, respectively, vs. D0) (Figure 3E), and significant upregulation of *P75* (D0 vs. D1, 3, 5, 7, ****P < 0.0001) (Figure 3F) with progression of the degeneration. Neuronal marker and intermediate filament *Nestin*, showed transient significant upregulation at D1 post-harvest (****P < 0.0001), followed by return to baseline levels at later time points (Figure 3G).

The analysis of neurotrophic factors expression in our model showed upregulation of the key neurotrophins such as *Ngf*, *Bdnf* and *Gdnf*. *Ngf* expression after *in vitro* WD was biphasic, with significant surge of *Ngf* expression at D1 and D5 (****P < 0.0001) compared to D0 samples (Figure 3H). The upregulation of *Bdnf* was gradual during the progression of WD, and significantly higher at D3 (*P < 0.05), D5 and D7 (****P < 0.0001) post-harvest, compared to D0 healthy nerve (Figure 3I). Furthermore, *Gdnf* expression levels were significantly increased at the earliest time point (D1, ****P < 0.0001), and gradually declined at D3 and 5 (**P < 0.01), to finally reach baseline levels at D7, all compared to D0 healthy nerve (Figure 3J). We also analysed at *Ngf* receptor *Trka* and noticed gradual downregulation of gene expression after WD induction, with levels significantly decreased at D5 (***P < 0.001) and D7 (****P < 0.0001) compared to D0 intact nerve (Figure 3K).

Finally, the gene expression of myelin proteins *P0*, *Pmp22* and *Mbp* was significantly downregulated following WD as early as the day after the harvest. Indeed, *P0* expression significantly decreased at D1 following degeneration (***P < 0.001), and was almost absent at D3, 5 and 7 (****P < 0.0001) (Figure 3L). Similarly, *Mbp* expression levels were significantly downregulated at D1 (**P < 0.01), and nearly undetectable at D3, 5, and 7 (****P < 0.0001) (Figure 3M). In addition, expression of *Pmp22* gene was significantly decreased at D1 (****P < 0.0001) and completely abolished at later time points (****P < 0.0001) (Figure 3N).

### Expression Changes of Key Proteins Confirm Features of WD

To evaluate the extent to which our model recapitulates WD, we assessed the expression of some of these genes at the protein level.

**FIGURE 4:**
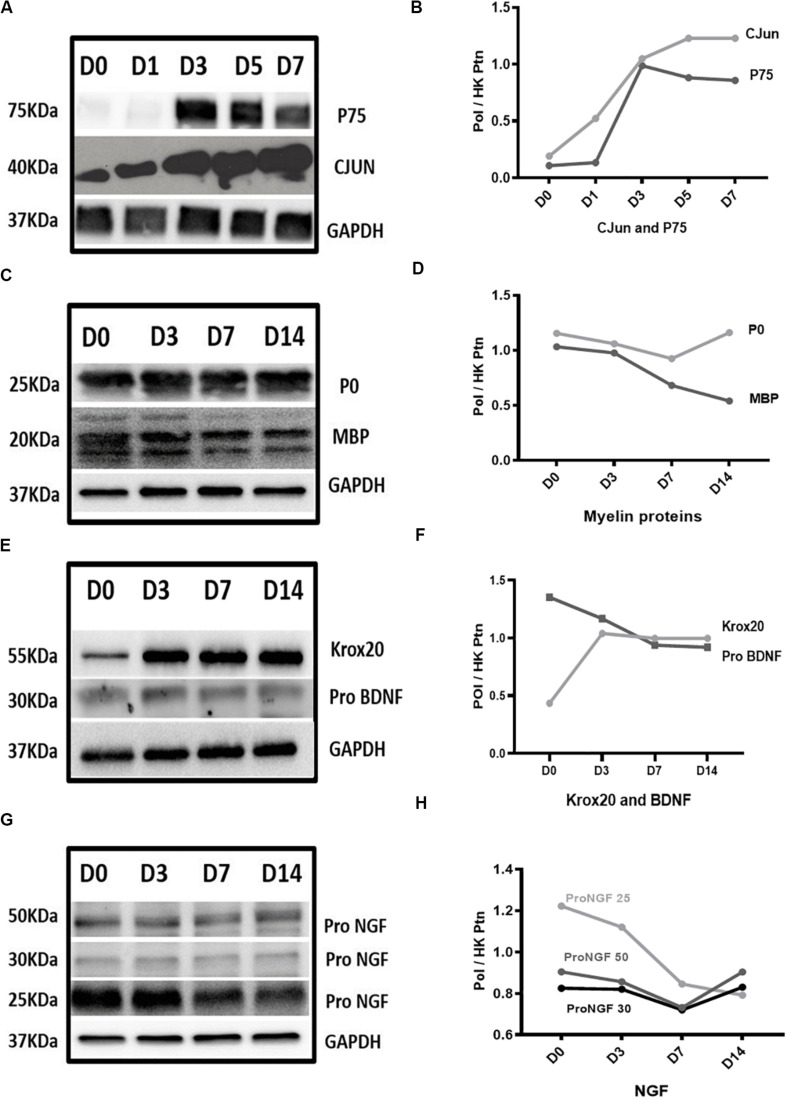
Protein expression results in Wallerian degeneration *in vitro* model. **(A,B)** Western blot images and graph, respectively, showed persistent upregulation of CJUN and P75 proteins after WD induction. **(C,D)** Western blot images and graph, respectively, showed gradual downregulation of MBP protein reaching approximately 50% of its expression at D14 and P0 remained constant throughout the 14 days of *in vitro* WD compared to D0 healthy nerve. **(E,F)** Western blot images and graph, respectively, showed upregulation of KROX20 protein at D3 post-degeneration reaching a plateau which remained constant until D14 post-degeneration. PRO-BDNF protein expression showed gradual downregulation starting from D3 post-regeneration and reaching 60% of expression at D14 compared to D0 control. **(G,H)** Western blot graph and image of PRO-NGF, respectively, showed gradual reduction of different isoforms gradually reaching almost the same level at D14. Data is normalised by GAPDH for each time point. (PoI) is Protein of Interest. (HK ptn) is house-keeping protein.

We observed upregulation of both CJUN and P75 proteins after *in vitro* WD with levels reaching up to seven fold and six fold, respectively, at D7 compared to D0 healthy nerve (Figures 4A,B). MBP protein showed gradual downregulation resulting in approximately 50% of expression at D14, compared to D0 healthy nerve. Conversely, P0 protein expression remained constant throughout the 14 days of the *in vitro* WD model (Figures 4C,D). KROX 20 protein expression levels raised at D3 post-degeneration, reaching a plateau which remained constant until D14 post WD induction (Figures 4E,F). PRO-BDNF protein expression showed an opposite trend with the neurotrophin gradually downregulated starting from D3 post degeneration and reaching 60% expression at D14 compared to D0 controls (Figures 4E,F). In addition. PRO-NGF protein expression gradually decreased: interestingly, amongst the PRO-NGF isoforms, the 25 kDa variant showed the greatest decrease in expression levels at D14 post degeneration compared to D0 control (Figures 4G,H).

### Assessing Novel Stem Cell Therapies With the *in vitro WD* Model: A Proof of Principle

**FIGURE 5:**
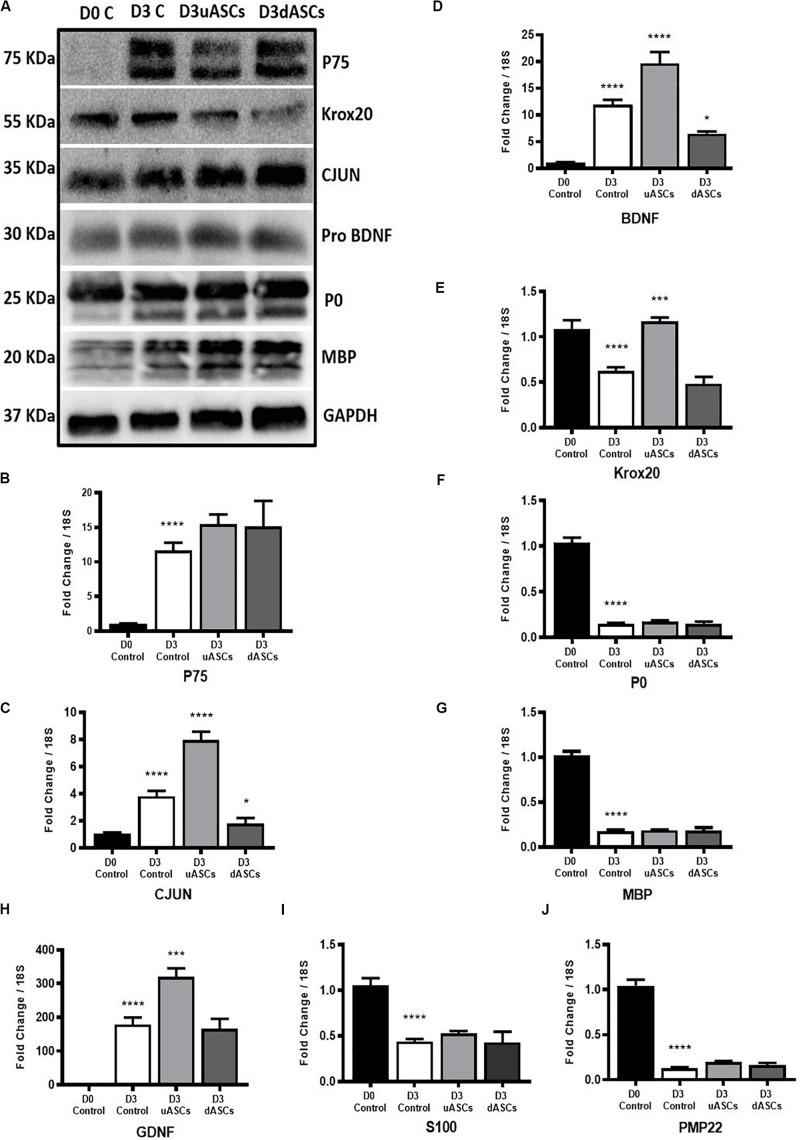
Gene and protein expression results of key transcription factors, Schwann cell markers, neurotrophic factors and myelin proteins of Wallerian degeneration model application. Indirect co-culture with both undifferentiated and differentiated adipose stem cells (uASCs, dASCs). **(A)** Protein expression of P75, KROX20, CJUN PRO-BDNF, P0 and MBP showing: an increase in the expression of P75 by five-fold at D3 without intervention and almost the same folds increase with AD-MSCs intervention compared to D0 control. An increase in expression of CJUN at D3 without intervention and continued upregulation by 30% for AD-MSCs and 50% fold for dASCs intervention compared to D0 control. Protein expression of KROX20 showing: upregulation of KROX20 at D3 without intervention with 30% and 50% downregulation at both AD-MSCs and dASCs intervention, respectively, compared to D0 control. No change in PRO-BDNF expression at D3 control, AD-MSCs and dASCs intervention compared to D0 control. Protein expression of myelin proteins showing: persistence of P0 and MBP proteins at D3 control and after stem cell intervention. **(B)** *P75* gene expression is upregulated in all D3 groups compered to D0 control that is further potentiated with both AD-MSCs and dASCs intervention. **(C)** *Cjun* gene expression is upregulated at D3 control group and AD-MSCs intervention markedly upregulates it gene expression in uASCs and dASCs were compared regarding D3c not D0 control whereas dASCs downregulates *Cjun* transcripts compared to D3 control levels **(D)** *Bdnf* gene expression is upregulated in all D3 groups compared to D0 control. AD-MSCs further potentiates this upregulation while dASCs intervention supresses it compared to D3 control group. **(E)** *Krox20* gene expression is downregulated at D3 non-interventional control group there is marginal downregulation with with dASCs intervention compared to D3 control; however, significant upregulation is noticed with AD-MSCs intervention compared to D3 control. **(F)** Significant downregulation of *P0* gene in all D3 groups compared to D0 control **(G)** Significant downregulation of *Mbp* gene in all D3 groups compared to D0 control. **(H)** The *Gdnf* gene is upregulated in all D3 groups compared to D0 control. Relative to D3 non-interventional control, AD-MSCs upregulates *Gdnf* levels but no effect is observed in the dASCs group. **(I)** *S100* gene expression is significantly downregulated to roughly about the same degree in nerves in all D3 groups compared to D0 control. **(J)** *Pmp22* gene expression is significantly downregulates in all D3 groups compared to D0 control. Gene data is presented as mean ± SEM (error bars) by one-way ANOVA (Tukey’s multiple comparison test) (*P < 0.05, **P < 0.01, ***P < 0.001, ****P < 0.0001).

To test the efficacy of our model to evaluate the potential of novel therapeutic approaches for nerve regeneration, we tested stem cell intervention with degenerating nerve explants. The nerve explants were co-cultured indirectly with AD-MSC or dASCs for 3 days; we then performed analysis of both gene and protein expression for the key markers of WD previously characterised (Figure 5).

We confirmed upregulation of P75 gene/protein following 3 days of WD (****P < 0.0001), compared to D0 (control); nevertheless, AD-MSC and dASC intervention only marginally increased *P75* expression at the gene level compared to D3 control (Figure 5B), and did not seem to affect protein levels (Figure 5A). Expression of *Cjun* gene was also significantly upregulated at D3 of WD (****P < 0.0001) compared to D0 (control); AD-MSC intervention further increased the gene expression of *Cjun* (*P* < 0.0001), whereas dASCs contributed to a downregulation of the *Cjun* transcripts to D3 control levels (*P < 0.05, Figure 5C). At protein levels, we observed upregulation of CJUN in all D3 groups, with a 50% increase in the dASCs groups compared to D0 control (Figure 5A). Similarly, *Bdnf* gene expression is significantly increased following WD compared to D0 (****P < 0.0001), further upregulated by co-culture with AD-MSC (****P < 0.0001), but significantly decreased following dASCs intervention compared to D3 control (*P < 0.05, Figure 5D). On the other hand, the levels of expression of PRO-BDNF proteins were largely unaltered in all experimental groups (Figure 5A).

As shown during baseline characterisation of the *in vitro* model, we confirmed that *Krox20* gene expression is significantly downregulated following WD (****P < 0.0001), whereas protein levels are increased at D3 (Figures 5A,E). AD-MSC intervention caused a significant upregulation of *Krox20* transcript compared to D3 control (***P < 0.001) which did not translate to an increase in protein levels (Figures 5A,E). Conversely, co-culture to dASC did not significantly alter gene expression levels of *Krox20*, which was comparable to D3 control (Figures 5A,E). All myelin genes were found to be downregulated following the *in vitro* degeneration, and stem cell interventions did not alter the gene expression levels (Figures 5F,G,J for *P0*, *Mbp* and *Pmp22*, respectively). This was not the case at protein level, where expression of P0 and MBP was sustained despite *in vitro* WD and AD-MSC/dASCs intervention (Figure 5A). We confirmed the increase in *Gdnf* gene expression at D3 post WD (****P < 0.0001) with further significant upregulation in the AD-MSC co-culture group compared to D3 control (***P < 0.001, Figure 5H), but no effect was observed in the dASCs group. The gene expression levels of the SC marker *S100* showed significant decline at D3 control and no changes after AD-MSC/dASC intervention compared to D3 control (Figure 5I).

## Discussion

The vast majority of scientific endeavour in the field of peripheral nerve injury focuses on the regenerative response of nerves, including stem cell interventions. Given that efficient degeneration of the distal stump is a prerequisite for regeneration, it is an important target for interventions aimed at improving nerve regeneration outcomes. Studies on WD have predominantly been performed *in vivo* which incorporates the local response of SCs and resident macrophages with the more complex systemic pathophysiological immune response following injury. Establishing a model for WD, in which the immune response and other systemic influences are excluded is appealing because such a model would permit the exploration of novel therapies such as stem cell intervention, in higher throughput *in vitro* systems and focus on local effects which can otherwise be lost. Although the concept of using nerve segments *in vitro* as a representative of WD model was adopted previously, the cut nerve segments were allowed to degenerate *in vitro* for only 48 h without characterisation; moreover, the nerves were not de-sheathed, a condition that allows cells other than SCs to engage in the degenerative response and influences the cell population being characterised (Fex Svenningsen and Kanje, 1998). In this work, we have characterised an *in vitro* model of WD allowing better understanding of the biological changes that take place within the nerve without systemic influence. Our model clearly demonstrates histoarchitectural degeneration and gene/protein expressions in keeping with significant degeneration of the nerve and switching on of the SC regenerative response. In addition, as a proof of principle, we have used the model to study the effect of AD-MSC intervention in WD. Day 3 was selected for study of this intervention for two reasons: (i) many of the WD events are underway with morphological and molecular changes clearly evidenced in our model; and (ii) it is hypothesised that the impact of stem cell intervention is likely to be early before the onset of transplanted cell death.

### Transcription Factors

Our *in vitro* model identified early and persistent upregulation of CJUN protein after injury. There is strong evidence that the transcription factor CJUN is a key reprogramming factor modulating the activity of repair SCs (Parkinson et al., 2008; Glenn and Talbot, 2013; Boerboom et al., 2017). CJUN drives increase in the expression levels of a multitude of trophic factors that are necessary for nerve regeneration acting both directly, such as GDNF and artemin, and indirectly, BDNF and NGF (Fontana et al., 2012). Gene ontology analyses have shown that *Cjun* modulates the expression of 172 genes, all involved in neuronal growth and regeneration (Arthur-Farraj et al., 2012). In our *in vitro* model of WD *Cjun* gene expression is upregulated at day 1 post injury but returns to normal over the 7 day time course; however, its levels are sufficient to induce persistent elevation of its protein levels which is the ultimate goal to trigger its cascade of influence in particular negative regulation of myelination and upregulation of neurotrophic factors. It is worth mentioning that upregulation of CJUN after nerve injury has been recently observed in human degenerating nerves, an observation that is comparable to our rodent *in vitro* model (Wilcox et al., 2020).

Mature SCs constitutively express SOX10 (Britsch et al., 2001; Jessen and Mirsky, 2019) which maintains expression of ErbB3 receptors for NRG1 and regulates *Oct 6* and *Krox20* in controlling myelination (Pereira et al., 2012). Whilst *Sox10* gene expression levels are unchanged following injury, our *in vitro* model demonstrates that this regulatory pathway is interrupted after nerve injury leading to a dramatic decrease in the level of both *Oct6* and *Krox20* genes across all time points after nerve injury induction. Although KROX20 protein is present at D14, the changes in gene expression of these transcriptional factors are in line with *in vivo* models of WD (Jessen and Arthur-Farraj, 2019). The accumulated KROX20 protein in our model may be attributed to the lack of systemic inflammatory response that enhances the capability SCs to remove cell debris and non-functioning proteins. Furthermore, the expression of *S100*, a pan SC marker, is rapidly reduced and does not return for at least 7 days in our model and in previous *in vivo* models (Perez and Moore, 1968). Together, the expression patterns of these key SC mediators confirms the reliability of our model to be used as a simulator to the previously described *in vivo* models of WD.

With the intervention of human AD-MSC to the *in vitro* WD model system, we noticed a promising effect of human dASCs increasing the upregulation of CJUN protein to 1.5-fold increase at D3 compared to D3 control; and downregulation of both gene and protein expression of KROX20. This significant enhancement of transcriptional control could result in a rapid switch off of pro-myelinating pathways which may result in a more rapid deployment in greater scale of the ‘repair’ SCs in their critical roles in WD.

### Neurotrophic Factors

Our *in vitro* WD model produces neurotrophic factor expression largely in keeping with that observed in *in vivo* models of injury. SCs upregulate neurotrophic factor expression and their cell surface receptors to prevent the death of injured neurons and stimulate axonal regeneration (Jessen and Arthur-Farraj, 2019).

In keeping with gene expression changes in our model, *Bdnf* expression has been demonstrated to increase up to 28 days post rat sciatic nerve transection (Meyer et al., 1992; Funakoshi et al., 1993); *Gdnf* upregulated progressively to day 7 post injury (Naveilhan et al., 1997); *Ngf* had a biphasic pattern of upregulation after nerve injury with a rapid surge within 6 h followed by sharp decrease and subsequent increase again by day 3 with persistent elevation up to 14 days post transection (Heumann et al., 1987a; Meyer et al., 1992); and upregulation of the neurotrophin receptor *P75*^*ntr*^ (You et al., 1997; Boyd and Gordon, 2003). It is difficult to compare the temporal relationship of these models too precisely with our own given the nature of the injury differs from crush to single cut as opposed to our own double level transection; however, it is clear that the systemic immune response to injury will play an important role in neurotrophic factor expression, in particular *Ngf* via IL-1β. Macrophage invasion occurs 3 days after injury which is correlated with the second surge of *Ngf* levels in the *in vivo* model. This observation is further accredited *in vitro* by addition of macrophages or IL-1β (Heumann et al., 1987b). However, in our model SCs achieved a delayed second surge of *Ngf* gene expression at day 5 without the systemic inflammatory triggers.

Protein expression of the neurotrophic growth factors in our model decreases over time which is likely as a result of increased growth factor secretion, although we did not measure this directly. NGF protein has different isoforms with distinct molecular weights and is secreted as a PRO-NGF precursor biologically active on differing tissues (Fahnestock et al., 2004). PRO-NGF 25 protein expression was most significantly depleted in our model over 14 days post WD induction implying gradual secretion of this mature NGF. It is also notable that the P75 receptor increases by day 3 and remains at this level for at least 7 days.

AD-MSC have the ability to improve neurite outgrowth *in vitro* and *in vivo* which is likely as a result of neurotrophic factor expression (Faroni et al., 2014; Kingham et al., 2014). In our *in vitro* WD model, the upregulation of *Bdnf* and *Gdnf* in SCs was significantly greater after co-culture with AD-MSC than compared to control degenerated nerve explants at D3. This provides further evidence that AD-MSC can potentiate SCs into a regenerative phenotype. Interestingly, dASC failed to produce this response in SCs which may be attributable to the de-differentiation of dASC following growth factor withdrawal (Faroni et al., 2016).

### Myelin Features in Our *in vitro* Model

Following peripheral nerve injury, the adaptive injury response triggered in SCs results in loss of axonal contact and myelin maintenance is switched to clearance initiated by SC myelinophagy (Gomez-Sanchez et al., 2015; Jang et al., 2016). In accordance with this, myelin protein gene (*P0, Mbp and Pmp22*) expression was switched off and there was progressive morphological disorganisation and degradation of myelin protein and lipid structures following injury in our *in vitro* WD model.

Using TEM and immunohistochemistry on the injured nerve *in vitro* over 2 weeks, we noted the early appearance of multiple vacuoles and myelin lamellae collapse, followed by axonal degeneration and complete myelin disorganisation in keeping with previous findings (Johannessen, 1979; King, 1999; Kidd et al., 2013); however, myelin debris persisted in our model and this may be an important attribute to explore further because myelin debris inhibits pathways for axonal regeneration (Boerboom et al., 2017). SCs work in combination with resident and haematogenous macrophages toward myelinophagy, an active macro-autophagic process induced by SCs after injury (Gomez-Sanchez et al., 2015; Jang et al., 2016). In non-desheathed nerves, we noted an increase in CD68 staining for macrophages at days 7 and 14. These are presumed to be epineurium resident macrophages responding to the inflammatory process initiated by repair SCs although the systemic response describes invasion of macrophages by day 3 (Mueller et al., 2001). In our desheathed model, however, the epineurium is removed which likely removes the majority of resident macrophages although internal connective tissue structure is maintained. The persistence of myelin proteins at 14 days reveals one significant difference between our model and other systems either *in vivo* or the culture of dissociated SCs in which myelin degradation occurs (Gomez-Sanchez et al., 2015).

The importance of the systemic immune response in particular macrophages has been described as mandatory for complete and effective WD and myelin clearance (Scheidt et al., 1986). In a model of *in vivo* WD in which the mouse nerve explants were embedded in chambers with differential pore sizes to permit or exclude immune cells. Nerves exposed to macrophages and leucocytes permitted complete degeneration of myelin, whilst the lack of immune cells resulted in persistent myelin debris at 28 days. Furthermore, SCs secrete and activate pro-inflammatory markers after WD induction, such as TNFα and interleukin which lead to disturbance of the blood nerve barrier and allow monocytes and haematogenous macrophages to reach the field (Gaudet et al., 2011). Toll like receptors (TLRs) are expressed on the surface of SCs after injury and full knockout of TLRs in injured nerve leads to retardation in myelin clearance and macrophage recruitment (Chen et al., 2015). We can conclude that our *in vitro* model, in which there is no systemic inflammatory or immune response to aid SCs in myelinophagy, succeeded in early myelin collapse and breakdown but failed to achieve myelin clearance. There is promising pharmaceutical research targeting WD in many disorders of the central and peripheral nervous system (Coleman and Hoke, 2020). This leaves the *in vitro* WD model open to controlled manipulation of the key immune players in an *in vitro* system; in addition to the investigation of interventions such as pharmaceutical agents and stem cells to activate or inhibit autophagy machinery in SCs.

## Data Availability Statement

All datasets presented in this study are included in the article/supplementary material.

## Ethics Statement

The studies involving human participants were reviewed and approved by National Research Ethics Committee (NRES 13/SC/0499). The patients/participants provided their written informed consent to participate in this study. Ethical review and approval was not required for the animal study because only *ex vivo* animal tissue was used. All the animal work was performed in accordance with the UK Animal Scientific Procedures Act 1986.

## Author Contributions

HbE, AF, MA, JO, and LW performed all studies and analysis. HbE, HsE, MM, AZ, and AR conceived of the study and drafted the manuscript. All authors read and approved the final manuscript.

## Conflict of Interest

The authors declare that the research was conducted in the absence of any commercial or financial relationships that could be construed as a potential conflict of interest. The reviewer VM declared a past co-authorship with one of the authors AF to the handling Editor.

## Abbreviations

AD-MSCs: adipose derived mesenchymal stem cells
aMEM: alpha-modified minimum essential Eagle’s medium
BDNF: brain derived neurotropic factor
DAPI: 4’,6-diamidino-2-phenylindole
dASCs: differentiated adipose stem cells
DMEM: glucose Dulbecco’s Modified Eagle’s medium
FBS: foetal bovine serum
GAPDH: glyceraldehyde 3-phosphate dehydrogenase
GDNF: glial derived neurotropic factor
HBSS: Hanks balanced salt solution
IHC: immunohistochemistry
MBP: myelin basic protein
NGF: nerve growth factor
OCT: optimum cutting temperature
P0: myelin protein zero
PBS: phosphate-buffered saline
PCR: polymerase chain reaction
PFA: paraformaldehyde
PMP22: peripheral myelin protein 22
RNA: ribonucleotide acid
RT: room temperature
SCs: Schwann cells
SVF: stromal vascular fraction
TEM: transmission electron microscope
TLRs: toll like receptors
WD: Wallerian degeneration.
